# Umbilical Nodule with Cyclical Bleeding: A Case Report and Literature Review of Atypical Endometriosis

**DOI:** 10.1155/2016/7401409

**Published:** 2016-09-22

**Authors:** Marlene Teixeira Andrade, Cláudia V. Marques de Freitas, Sara Filipa Camacho Câmara, José Joaquim Nunes Vieira

**Affiliations:** ^1^Centro Hospitalar de Trás-Os-Montes e Alto Douro, Unidade de Vila Real, Avenida Noruega, 5000-508 Vila Real, Portugal; ^2^Hospital Dr. Nélio Mendonça, Hospital Central do Funchal, Avenida Luís de Camões, Funchal, 9004-514 Madeira Island, Portugal

## Abstract

Endometriosis is defined as the presence of endometrial glands and stroma outside the uterus. It affects 3 to 10 percent of women of reproductive age. Umbilical endometriosis is rare, with an estimated incidence of 0.5–1.0% among all cases of endometriosis, and is usually secondary to prior laparoscopic surgery involving the umbilicus. In this report, we described a case of umbilical endometriosis treated with surgical resection and highlight the great importance of medical history compared to complementary diagnostic tests that can be sometimes inconclusive.

## 1. Introduction

Endometriosis is a common benign disease, which is defined by the presence of endometrial tissue outside the uterus [1]. The first description of this condition was made by a German physician, Daniel Schroen, in 1690 [2]. It affects 3 to 10 percent of women of reproductive age [3, 4] and represents an important cause of infertility [5]. It occurs in 2–22% of asymptomatic patients, in 20–30% of infertility cases, and in 40–60% of dysmenorrhoea cases [6]. Its etiopathogenesis is not well established yet. Endometriosis can affect any woman from premenarche [7] until postmenopause, irrespective of the race, ethnicity, or maternal status [8, 9]. It is reported that up to 70% of adolescents with chronic pelvic pain before menarche can be affected with endometriosis [7]. The most frequent locations include the ovaries, the uterosacral ligaments, the pouch of Douglas, and the other pelvic organs. Extraperitoneal locations as cervix, vagina, vulva, lungs, umbilicus, or postoperative scars are uncommon [6] and locations like nasal mucosa, brain, and eyes are very rare [10]. There is little evidence on the real incidence and prevalence of these extrapelvic lesions. Umbilical endometriosis rarely occurs, with an estimated incidence of 0.5–1.0% among all cases of endometriosis [11, 12], usually affecting patients after laparoscopy or other surgical procedures involving the umbilicus [4] (of the reported cases of cutaneous endometriosis, over 70% are secondary to prior surgery and occur at the site of surgical scars) [13, 14]. Umbilical endometriosis can be classified as primary, named Villar's nodule, when it appears spontaneously (any ectopic endometrium that is found superficial to the peritoneum without any history of previous surgery) or secondary, when it appears after surgical procedures. The term secondary endometriosis can be used even when it is not located on surgical scars, such as on the umbilicus, but only if its onset occurs within 2 years after the procedure [15–17]. The differential diagnosis of umbilical tumors in women comprise endometriosis in 32.2%, benign primary tumor in 29.7%, metastatic tumor in 29.7%, and malignant primary tumor in 8.4% [12, 18]. The maximal depth of penetration of the umbilical endometriosis described is up to the fascial level [19–21].

To treat umbilical endometriosis, wide resection with 2 mm margins is generally recommended [22] and there are few cases in which conservative treatment is indicated [23].

The authors report a case of a patient with umbilical endometriosis associated with a previous laparoscopic intervention and treated by surgical excision.

## 2. Case Presentation

**Figure 1:**
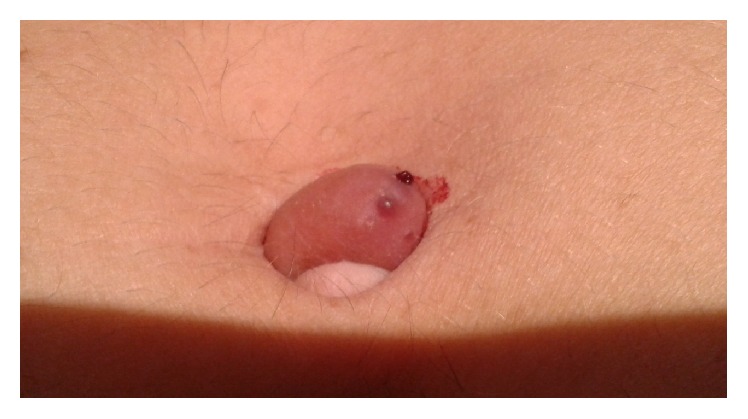
Umbilical nodule bleeding during menstrual cycle.

**Figure 2:**
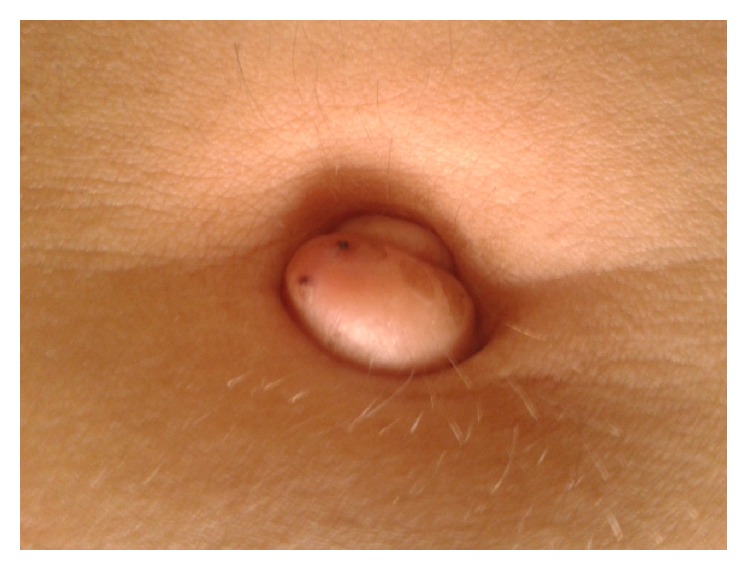
Umbilical nodule.

A forty-two-year-old healthy female, with menarche at the age of 13, was referred to the gynecology department by general surgery, with a livid coloured nodule in the umbilicus which gradually increased in size over the past 3 years. She also presented with dysmenorrhoea (numeric rating scale of pain (NRS): 10), dyspareunia (NRS: 10), dyschezia (NRS: 7), and tenesmus. She was medicated with an oral contraceptive with ethinylestradiol and gestodene. The nodule was painless and the patient mentioned cyclical umbilical bleeding synchronized with menstruation (Figure 1), during withdrawal bleeding. She had irregular menstruation periods and a cesarean for fetal breech presentation 9 years before. The patient had past history of laparoscopic appendectomy five years previously, with umbilical cannulation. The histopathological examination of the appendix revealed endometriosis. She has no known family history of endometriosis. At physical examination, she had a soft swelling nodule, with two bluish-purple dots, in the umbilicus with a diameter of 1.2 cm, with a normal skin envelope, that was irreductible (Figure 2). In this first gynecological consultation, the hormonal medication was changed to dienogest 2 mg continuously. The patient stayed in amenorrhea with no bleeding of the umbilical nodule.

**Figure 3:**
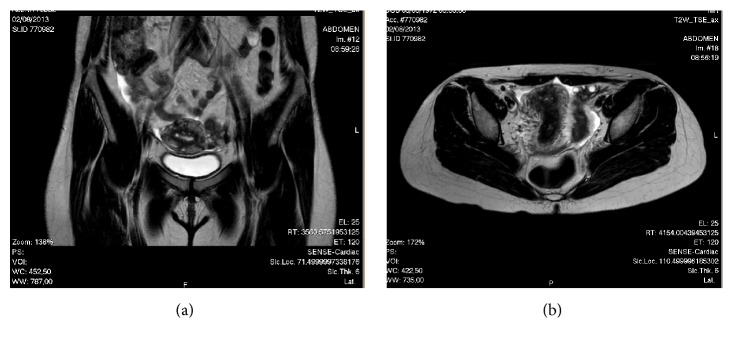
MRI: T2-weighted imaging. (a) Coronal view. (b) Axial view: uterus with multiple small nodules regarded as leiomyomas and with some endometrial glands suggesting adenomyosis.

**Figure 4:**
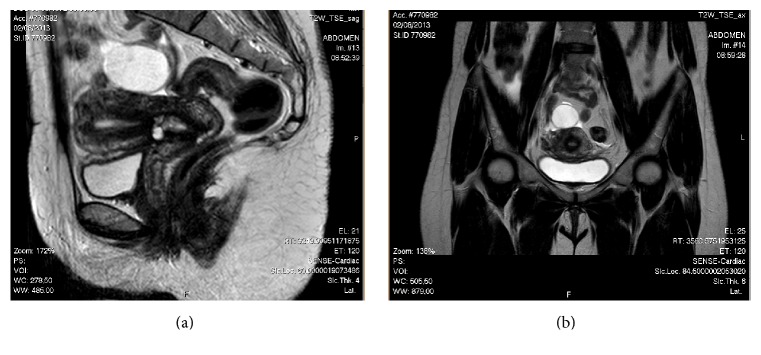
MRI: T2-weighted imaging. (a) Sagittal view. (b) Coronal view: left ovarian cyst.

Abdominal examination was otherwise normal with no clinical signs of hernia. The first ultrasonography of the umbilical nodule revealed an image suggestive of dermoid cyst. Pelvic computed tomography (CT) scan revealed two contiguous cystic images with peripheral contrast enhancement in the left adnexal area that were included in the differential diagnosis of endometrioma, tubo-ovarian abscess, and serous cystadenoma. A second ultrasound scan of the lesion revealed two superficial cysts measuring 2 mm and another deeper cyst measuring 4 mm, with nonspecific aspect, though compatible with sebaceous or dermal inclusion cysts. Magnetic resonance imaging (MRI) showed uterus measuring 92 × 40 × 58 mm, with multiple small nodules showing signal hypointensity on T2-weighted imaging regarded as intramural leiomyomas and the presence of some endometrial glands suggesting adenomyosis, endometrium with 6 mm (Figure 3). MRI also revealed a left septated ovarian cyst measuring 40 × 33 mm and a second cyst of 28 × 18 × 24 mm, containing fluid showing signal hyperintensity on T1-weighted imaging and slightly signal hyperintensity on T2-weighted imaging, suggesting hemorrhagic cyst or endometrioma (Figure 4).

**Figure 5:**
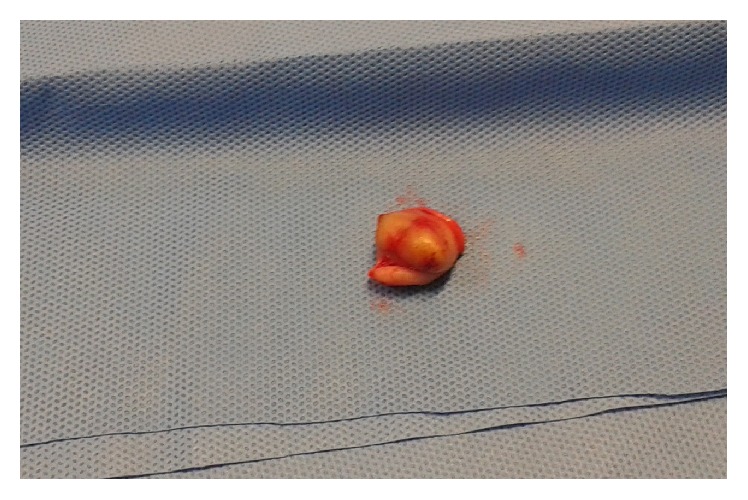
Umbilical nodule resected.

The patient underwent excision of the nodule under general anesthesia. The nodule was supra-aponeurotic with central extension (hernial ring) to the abdominal peritoneum (Figure 5). Diagnostic laparoscopy was also performed and intraoperative findings showed several typical endometriosis spots in the vesicouterine fold, pelvic peritoneum, and posterior fornix, increased right ovary measuring 3 cm with a simple cyst of 2.5 cm, and an endometrioma measuring less than 1 cm. Cauterization of endometriotic lesions with bipolar energy and spontaneous drainage and coagulation of the capsule of the endometrioma were performed. She was discharged from hospital after 24 hours and medicated with desogestrel on a continuous basis.

The patient was observed at 1, 3, 6, and 12 months after surgery and there was no evidence of clinical recurrence 13 months after that. The hormonal medication was then changed to dienogest plus ethinylestradiol and the patient remained asymptomatic. Histology of the lesion showed the presence of endometrial glands and stroma; therefore a diagnosis of umbilical endometriosis was made. The clinical evaluation for follow-up will be done annually.

## 3. Discussion

Endometriosis is a common disorder, defined as the extrauterine presence of endometrial glands and stroma. It is a common, estrogen-dependent inflammatory disease, affecting 3–10% of women in the reproductive age [24, 25]. Endometriosis is mostly located in the pelvis but can also be encountered at nearly any organ of the body, as the lungs, bowel, ureter, brain, inguinal canal, and abdominal wall [3, 26–28]. The symptoms most frequently associated with endometriosis are pelvic pain, dysmenorrhoea, dyspareunia, and subfertility/infertility [24, 25, 29]. Its pathogenesis is not completely understood and multiple theories have been proposed to explain it: implantation of endometrial cells through retrograde menstruation (endometrial tissue is transported during menstruations from the uterus through the fallopian tubes, therefore gaining access to and implanting on pelvic structures (the implantation or retrograde menstruation theory) [3]), haematogenous or lymphatic dissemination of endometrial cells (the dissemination theory), ectopic differentiation of pluripotent peritoneal progenitor cells to endometrial tissue (coelomic metaplasia theory) [3, 24, 25, 30], production of substances to form endometriosis by sloughed endometrium (the induction theory), specific stimulation to a Müllerian origin cell nest producing endometriosis (the embryonic rest theory), and proliferation of ectopic endometrial cells produced by alterations in cell-mediated and humoral immunity (the cellular immunity theory) [3].

The most common locations of endometriosis are the ovaries (up to 88% of all cases), followed by the appendix, intestine, cervix, omentum, and skin [2]. In 70% of cutaneous endometriosis, it is more frequently secondary, following abdominopelvic surgery, but can appear spontaneously (30%), when in the absence of prior surgery [2]. In the latter case, it appears most commonly on the umbilicus, followed by the inguinal region [2, 11, 12], and there are cases in which the lateral abdominal wall is involved [3, 31, 32].

The pathogenesis of primary umbilical endometriosis is still unclear. Possible explanations for this disorder could be the migration of endometrial cells to the umbilicus through the abdominal cavity, the lymphatic system, or through the embryonic remnants in the umbilical fold such as the urachus and the umbilical vessels [12, 18], genetic predisposition, and immunological defects [6]. The most feasible explanation for secondary umbilical endometriosis is the* direct transplantation theory* which says that this type of endometriosis is caused by iatrogenic dissemination of endometrial cells, for example, a laparoscopic operation [3]. Spontaneous cutaneous endometriosis is associated with more severe pelvic disease than scar endometriosis [12, 20].

Extragenital or extrapelvic endometriosis is even more difficult to diagnose due to the extreme variability in presentation [33]. Umbilical endometriosis presents as a rubbery or firm nodule, and the size can vary from several millimeters to 6 cm. A pathognomonic sign for this type of lesion is the umbilical sanguinolent discharges simultaneously with the menstruation period [6]. Clinical symptoms typically include pain, bleeding, and swelling concurrently with menstruation, but some patients are asymptomatic [2, 12]. Nevertheless, there are studies which describe the pain as constantly present and with no association with the menstrual cycle in most cases [3, 34]. The presence of concomitant abdominal masses or previous history of endometriosis is common (26% of cases) [24], frequently associated with complaints of dysmenorrhoea, dyspareunia, or defecation pain [2].

Numerous studies have reported a long delay in the diagnosis of endometriosis [35]. There are several symptoms which can be predictive of endometriosis as severe dysmenorrhoea in infertile women, abdominopelvic pain, dysmenorrhoea, heavy menstrual bleeding, infertility, dyspareunia, postcoital bleeding and/or previous diagnosis of ovarian cyst, irritable bowel syndrome, or pelvic inflammatory disease [36] and these symptoms and signs can help us to consider the diagnosis of endometriosis when an umbilical nodule is present. In order to diagnose this condition, CT, MRI, ultrasonography, and serum carbohydrate antigen 125 levels are commonly used, but the results of these screening procedures are inconclusive. Ultrasound is useful in defining whether a mass is cystic or solid, but it has low specificity for diagnosing endometriomas [3, 37]. The ultrasound characteristics of endometriomas, in premenopausal women, include ground glass echogenicity, one to four compartments, and no papillary structures with detectable blood flow [38]. In women with signs and symptoms of rectal endometriosis, transvaginal sonography is useful for detecting or ruling out rectal endometriosis [39], but as it is a procedure very dependent on the operator, it is not recommended for diagnosis of rectal endometriosis, except if it is executed by clinicians highly experienced in transvaginal ultrasound [35]. In umbilical endometriosis, CT scan generally demonstrates a solid well-circumscribed mass and may be useful in showing the extent of the disease [3]. MRI is useful to define the size and locations of lesions and to exclude the possibility of intraabdominal extension [12, 40]. MRI usefulness to diagnose peritoneal endometriosis is not well established [41]. The suspicion of the diagnosis of umbilical endometriosis is based on the clinical presentation, but the definitive diagnosis should always be confirmed by histological examination following biopsy or resection [12]. The histological features required for the diagnosis of endometriosis are the concomitant presence of two of the three following characteristics: endometrial-like glands, endometrial stroma, or hemosiderin pigment [3].

The treatment of choice for umbilical endometriosis is the excision en-bloc of the lesions with wide margins with a subsequent course of medication effective in inducing endometrial atrophy [42, 43]. The laparoscopic pelvic observation should be performed as 13%–15% of umbilical endometriosis cases are accompanied by pelvic endometriosis [42]. Drugs such as gonadotropin-releasing hormone agonist (GnRH) or contraceptive pill alone is insufficient and should not be done before surgery, but this medication can be helpful for the relief of symptoms resulting from pelvic endometriosis. Therefore, there is several hormonal therapies used for the relief of pain associated with endometriosis: hormonal contraceptives, progestogens and antiprogestogens, GnRH agonists and antagonists, and aromatase inhibitors are at present in clinical use. All hormonal drugs used to treat pain associated with endometriosis are effective in controlling the symptoms and there is no clear evidence that a treatment is superior to another; thus treatment should be individualized, considering patient preferences, side effects, efficacy, costs, and availability [35]. Recommendations to patients after laparoscopic surgery are based on the operative findings and results of surgery. The main goal of postoperative medical treatment is suppressing ovarian activity resulting in atrophy of endometriotic lesions. The success of this therapy is related to the total excision of the endometriotic lesions so that the recurrence of the disease could be minimised and the absence of pain maintained [44]. Recurrence is rare after complete excision of the lesion [2, 12, 20]. Goldberg and Bedaiwy report a case of recurrence of secondary umbilical endometriosis 7 months following excision of the lesion and this was successfully treated with silver nitrate cautery [45]. The recurrent lesions can result from incomplete excision of previous lesions or from* de novo* cells. Identifying risk factors for recurrence may permit the recognition of subgroups at risk for disease control [44]. Several risk factors have been proposed, but there is controversy about which one is more predictive for recurrence. Risk factors like history of endometriosis surgery, bilateral pelvic involvement of endometriotic lesions, left-sided endometrioma, tenderness, nodularity at cul-de-sac, postoperative high revised American Fertility Society (rAFS) scores, and younger age were considered [44]. Ghezzi et al. reported that the probability of endometriosis recurrence is lower when it is located only on the right side of the pelvis and the time to achieve pregnancy after surgery, in couples who tried to conceive, seems to be shorter when the endometriosis is localized in the right hemipelvis [46]. Malignant transformation has been reported in 0.3–1% of cutaneous endometriosis. The most frequent histological subtypes are endometrioid carcinoma, clear cell carcinoma, adenosarcoma, and serous adenocarcinoma [2, 12, 20]. There is a reported case of clear cell adenocarcinoma arising from umbilical endometriosis in a 60-year-old woman who underwent hysterectomy for a uterine myoma at the age of 38 and who denied cyclic bleeding at the site of an umbilical cutaneous nodule correlating with menses until the age of 48 [47]. There are no proven favorable results of hormonal therapy within 6 months after surgery, if it is prescribed with the exclusive purpose of improving the outcome of surgery for pain [48]; nonetheless this hormonal therapy could be prescribed for other indications, as contraception or secondary prevention.

Our case describes a woman in her reproductive age with secondary umbilical endometriosis, with cyclical pain and umbilical blood discharge during menstruation, with previous diagnosis of endometriosis in the appendix and a concomitant endometrioma in the right ovary. Initially, before the patient has been referred to the gynecology department, umbilical endometriosis was misdiagnosed and was considered a dermoid cyst. After laparoscopy, our patient was followed up with a standard gynaecological examination, the assessment of painful symptoms, and a TV-US scan that were performed at 1, 3, 6, and 12 months, and this will be repeated subsequently on a yearly basis. The differential diagnosis of an umbilical nodule includes inflammatory disorders (abscess, folliculitis, and omphalitis), other benign lesions (lipoma, haemangioma, inclusion cyst, urachal anomalies, and pyogenic granuloma), umbilical hernia, and malignant tumors (adenocarcinoma, sarcoma, melanoma, metastatic visceral carcinoma—Sister Mary Joseph nodule) [13, 30].

This case highlights the importance of medical history compared to those of complementary diagnostic tests which are sometimes inconclusive. Surgeons should always consider umbilical endometriosis in their diagnostic approach when confronted with umbilical nodules. The evidence of the results of diagnosis and the different options to treat extragenital endometriosis is limited. Clinicians may consider surgical removal of symptomatic extragenital endometriosis, and when this is not possible, medical treatment should be considered to relieve symptoms. More studies are necessary to conclude about the best method of diagnosis of umbilical endometriosis and the most appropriate recommendations to treatment and follow-up which include the best medication to prevent the recurrence of the disease.

## References

[1] Theunissen C. I., IJpma F. F. (2015). Primary umbilical endometriosis: a cause of a painful umbilical nodule. *Journal of Surgical Case Reports*.

[2] Kyamidis K., Lora V., Kanitakis J. (2011). Spontaneous cutaneous umbilical endometriosis: report of a new case with immunohistochemical study and literature review. *Dermatology Online Journal*.

[3] Papavramidis T. S., Sapalidis K., Michalopoulos N. (2009). Spontaneous abdominal wall endometriosis: a case report. *Acta Chirurgica Belgica*.

[4] Paramythiotis D., Stavrou G., Panidis S. (2014). Concurrent appendiceal and umbilical endometriosis: a case report and review of the literature. *Journal of Medical Case Reports*.

[5] Fancellu A., Pinna A., Manca A., Capobianco G., Porcu A. (2013). Primary umbilical endometriosis. Case report and discussion on management options. *International Journal of Surgery Case Reports*.

[6] Pariza G., Mavrodin C. I. (2014). Primary umbilical endometriosis (Villar's nodule)—case study, literature revision. *Chirurgia*.

[7] Gogacz M., Sarzyński M., Napierała R., Sierocińska-Sawa J., Semczuk A. (2012). Ovarian endometrioma in an 11-year-old girl before menarche: a case study with literature review. *Journal of Pediatric and Adolescent Gynecology*.

[8] Machairiotis N., Stylianaki A., Dryllis G. (2013). Extrapelvic endometriosis: a rare entity or an under diagnosed condition?. *Diagnostic Pathology*.

[9] Simoglou C., Zarogoulidis P., Machairiotis N. (2012). Abdominal wall endometrioma mimicking an incarcerated hernia: a case report. *International Journal of General Medicine*.

[10] Ata B., Ates U., Usta T., Attar E. (2005). Cervical Endometriosis, a case presenting with intractable spotting. *Medscape General Medicine*.

[11] Latcher J. W. (1953). Endometriosis of the umbilicus. *American Journal of Obstetrics & Gynecology*.

[12] Omori M., Ogawa T., Nara M., Hashi A., Hirata S. (2014). Umbilical endometriosis with giant degenerated uterine leiomyomas: a case report. *Gynecologic Oncology Case Reports*.

[13] Gin T. J., Gin A. D., Gin D., Pham A., Cahill J. (2013). Spontaneous cutaneous endometriosis of the umbilicus. *Case Reports in Dermatology*.

[14] Fernández-Aceñero M. J., Córdova S. (2011). Cutaneous endometriosis: review of 15 cases diagnosed at a single institution. *Archives of Gynecology and Obstetrics*.

[15] Jaime T. J., Jaime T. J., Ormiga P., Leal F., Nogueira O. M., Rodrigues N. (2013). Umbilical endometriosis: report of a case and its dermoscopic features. *Anais Brasileiros de Dermatologia*.

[16] Attia L., Ben Temime R., Sidhom J. (2010). A case of cutaneous endometriosis developed on an abdominal scar. *Tunisie Medicale*.

[17] Esteves T., Cabrita J., Coelho R., Vale E. (2010). Endometriose cutânea—a propósito de um caso clínico. *Dermatology Online Journal*.

[18] Barrow M. V. (1966). Metastatic tumors of the umbilicus. *Journal of Chronic Diseases*.

[19] Stojanovic M., Radojkovic M., Jeremic L. (2014). Umbilical endometriosis associated with large umbilical hernia. Case report. *Chirurgia*.

[20] Agarwal A., Fong Y. F. (2008). Cutaneous endometriosis. *Singapore Medical Journal*.

[21] Victory R., Diamond M. P., Johns D. A. (2007). Villar's nodule: a case report and systematic literature review of endometriosis externa of the umbilicus. *Journal of Minimally Invasive Gynecology*.

[22] Din A. H., Verjee L. S., Griffiths M. A. (2013). Cutaneous endometriosis: a plastic surgery perspective. *Journal of Plastic, Reconstructive and Aesthetic Surgery*.

[23] Saito A., Koga K., Osuga Y. (2014). Individualized management of umbilical endometriosis: a report of seven cases. *The Journal of Obstetrics and Gynaecology Research*.

[24] Arkoulis N., Chew B. K. (2015). An unusual case of asymptomatic spontaneous umbilical endometriosis treated with skin-sparing excision. *Journal of Surgical Case Reports*.

[25] Bulun S. E. (2009). Endometriosis. *The New England Journal of Medicine*.

[26] Dwivedi A. J., Agrawal S. N., Silva Y. J. (2002). Abdominal wall endometriomas. *Digestive Diseases and Sciences*.

[27] Tomás E., Martín A., Garfia C. (1999). Abdominal wall endometriosis in absence of previous surgery. *Journal of Ultrasound in Medicine*.

[28] Seydel A. S., Sickel J. Z., Warner E. D., Sax H. C. (1996). Extrapelvic endometriosis: diagnosis and treatment. *The American Journal of Surgery*.

[29] Mowad C., Andreychik C., Murphy T. (2014). Umbilical endometriosis elucidates cause of recurrent pneumothorax. *Journal of the American Academy of Dermatology*.

[30] Malebranche A. D., Bush K. (2010). Umbilical endometriosis: a rare diagnosis in plastic and reconstructive surgery. *Canadian Journal of Plastic Surgery*.

[31] Apostolidis S., Michalopoulos A., Papavramidis T. S., Papadopoulos V. N., Paramythiotis D., Harlaftis N. (2009). Inguinal endometriosis: three cases and literature review. *Southern Medical Journal*.

[32] Horton J. D., DeZee K. J., Ahnfeldt E. P., Wagner M. (2008). Abdominal wall endometriosis: a surgeon's perspective and review of 445 cases. *American Journal of Surgery*.

[33] Singh A. (2012). Umbilical endometriosis mimicking as papilloma to general surgeons: a case report. *Australasian Medical Journal*.

[34] Hensen J.-H. J., Van Breda Vriesman A. C., Puylaert J. B. C. M. (2006). Abdominal wall endometriosis: clinical presentation and imaging features with emphasis on sonography. *American Journal of Roentgenology*.

[35] Dunselman G. A. J., Vermeulen N., Becker C. (2014). ESHRE guideline: management of women with endometriosis. *Human Reproduction*.

[36] Ballard K. D., Seaman H. E., De Vries C. S., Wright J. T. (2008). Can symptomatology help in the diagnosis of endometriosis? Findings from a national case-control study—part 1. *BJOG: An International Journal of Obstetrics and Gynaecology*.

[37] Vincent L. M., Mittelstaedt C. A. (1985). Sonographic demonstration of endometrioma arising in cesarean scar. *Journal of Ultrasound in Medicine*.

[38] Van Holsbeke C., Van Calster B., Guerriero S. (2010). Endometriomas: their ultrasound characteristics. *Ultrasound in Obstetrics and Gynecology*.

[39] Hudelist G., English J., Thomas A. E., Tinelli A., Singer C. F., Keckstein J. (2011). Diagnostic accuracy of transvaginal ultrasound for non-invasive diagnosis of bowel endometriosis: systematic review and meta-analysis. *Ultrasound in Obstetrics and Gynecology*.

[40] Yu C.-Y., Perez-Reyes M., Brown J. J., Borrello J. A. (1994). Mr appearance of umbilical endometriosis. *Journal of Computer Assisted Tomography*.

[41] Stratton P., Winkel C., Premkumar A. (2003). Diagnostic accuracy of laparoscopy, magnetic resonance imaging, and histopathologic examination for the detection of endometriosis. *Fertility and Sterility*.

[42] Chikazawa K., Mitsushita J., Netsu S., Konno R. (2014). Surgical excision of umbilical endometriotic lesions with laparoscopic pelvic observation is the way to treat umbilical endometriosis. *Asian Journal of Endoscopic Surgery*.

[43] Buca D., Leombroni M., Falo E. (2013). Recurrence of primary umbilical endometriosis: case report and review of the literature. *Multidisciplinary Journal of Women's Health*.

[44] Selçuk İ., Bozdag G. (2013). Recurrence of endometriosis; risk factors, mechanisms and biomarkers; review of the literature. *Journal of the Turkish German Gynecological Association*.

[45] Goldberg J. M., Bedaiwy M. A. (2007). Recurrent umbilical endometriosis after laparoscopic treatment of minimal pelvic endometriosis: a case report. *Journal of Reproductive Medicine for the Obstetrician and Gynecologist*.

[46] Ghezzi F., Beretta P., Franchi M., Parissis M., Bolis P. (2001). Recurrence of ovarian endometriosis and anatomical location of the primary lesion. *Fertility and Sterility*.

[47] Obata K., Ikoma N., Oomura G., Inoue Y. (2013). Clear cell adenocarcinoma arising from umbilical endometriosis. *Journal of Obstetrics and Gynaecology Research*.

[48] Furness S., Yap C., Farquhar C., Cheong Y. (2004). Pre and post-operative medical therapy for endometriosis surgery. *The Cochrane Database of Systematic Reviews*.

